# Simulation examining the factors influencing capillary wick transport in a refrigerant direct cooling system for power battery packs

**DOI:** 10.1038/s41598-023-43457-4

**Published:** 2023-11-16

**Authors:** Yun Hu, Fengwu Shan, Jianbang Zeng, Shaohuan Liu, Zhengyuan Xing, Wenxiang Fu, Yufeng Luo

**Affiliations:** 1 Key Laboratory of Conveyance and Equipment, East China Jiaotong University, Ministry of Education, Nanchang, 330013 China; 2 School of New Science and Engineering, Xinyu University, Xinyu, 338004 China; 3 College of Automotive Studies, Tongji University, Shanghai, 20092 China; 4 Jiangxi Jiangling Group New Energy Automobile Co., Ltd, Nanchang, 330013 China; 5 School of Mechanical and Electronic Engineering Nanchang University, Nanchang, 330031 China; 6 Key Innovation Center of AI Industry and Higher Education Integration of Jiangxi Province, East China Jiaotong University, Nanchang, 330013 China

**Keywords:** Fuel cells, Computational science, Fluid dynamics

## Abstract

The effectiveness of power battery refrigerant direct cooling systems of electric vehicles incorporating capillary wicks is directly determined by these wicks’ transport performance. The Fries–Dreyer equation describes wicking behavior, but there is a significant gap between its predictions and the experimental results as reported in the literature. This work examines the factors influencing transport performance in an unconsolidated capillary wick with spherical particles. A mathematical and physical model is developed, the latter using the COMSOL software platform. Both the developed mathematical form and the numerically simulated results of this model are closer to the experimental results than those obtained using the Fries–Dreyer equation. The simulation results enable optimizing the equilibrium height and capillary time numbers providing a fitted Fries–Dreyer equation that is then used to analyze the influence of saturation, inclination angle, wick particle diameter, and tortuosity on the liquid rise mass and velocity and the equilibrium height, and the effects are in close but not perfect accord with experimental data. To narrow the gap, the Fries–Dreyer equation is further optimized using the numerically simulated results, substantially improving the accord with the experimental results.

## Introduction

The industrialized electric vehicle’s power battery thermal management system has experienced two generations of air- and liquid-cooling implementations since its development. Although the air-cooling system has the advantages of simple structure, low cost, easy maintenance, and so on, its expansion to the high-speed and fast charging in the electric vehicle market is limited due to the low heat transfer coefficient of air and the limited maximum heat dissipation capacity^1^. In contrast, the heat transfer medium in the liquid-cooling system has a higher heat transfer coefficient, which to some extent can meet the requirements of private cars and short-distance vehicles that have a low demand for fast charging^2^. However, the operation of long-distance electric vehicles generates a strong demand for fast charging; hence, there is an urgent need to develop a third-generation refrigerant direct cooling system for the power battery pack, to improve the environmental adaptability of such vehicles.

Direct contact cooling systems, where the refrigerant is in direct contact with the surface of the battery, have attracted wide attention because of their excellent temperature control performance and fast response rate. Al-Zareer et al.^3–7^ studied the battery thermal management system of hybrid electric vehicles with a pump-driven refrigerant (propane, ammonia, and R134a) and found that the height of the refrigerant in the battery pack, the inlet temperature of the refrigerant, the system saturation pressure, and the discharging and charging rates all had significant impacts on the maximum temperature and temperature differences throughout the battery pack. However, to ensure that the internal temperature of the battery pack is always in the comfortable operating temperature range (25–40 $\mathrm{{^\circ C}}$^8^) of lithium-ion batteries, the inlet propane, ammonia, or R134a should be liquid, resulting in a system pressure well above atmospheric that, so the system is prone to refrigerant leakage. Since the boiling point of fluoride in Novec 649 and Novec 7000 fluids is close to or falls within the comfortable temperature range for operation of lithium-ion batteries, an immersion cooling system of a lithium-ion battery with Novec 7000 as the refrigerant can greatly reduce the system pressure. This structure also provides a faster response rate than an air-cooled system and has good temperature control performance^9^. Wang et al.^10^ built a refrigerant direct cooling system for a power battery with a high-precision peristaltic pump driving Novec 7000, and considered the influence of the discharge rate, inlet velocity of the refrigerant, and the degree of subcooling on the cooling effect of the battery pack. In order to reduce the cost and complexity of the system, Hirokazu et al.^11^ used Novec 7000 and Novec 649 with the capillary material. They found that the temperature of the battery pack was effectively controlled when the discharge rate reached 20C, which improved system efficiency. We have extensively investigated various aspects of battery thermal management systems using a refrigerant direct cooling system based on capillary transport. Our research team found that the cooling effect of the battery pack depended on the transport mass (or volume), refrigerant velocity, and height of a capillary wick, and that the transport performance of a capillary wick was closely related to its pore structure^12^.

In recent years, related work in capillary wick plays a vital role, including the rise of groundwater in soil^13^, oil displacement by brine in the reservoir rock^14,15^, and water transport in plant tissue^16^. Scholars have also conducted a large amount of research to clarify the relationship between the transport performance of a capillary wick and its pore structure. Earlier, Lucas^17^and Washburn^18^ (who formulated Lucas-Washburn equation) found that the imbibition height and mass of water in a capillary wick constructed with a small cylindrical tube was related only to the capillary diameter. Later, Benavente et al.^19^ modified the Lucas-Washburn equation to determine the tortuosity of pore orientation and the roundness of the pore shape, and Rui et al.^20^ further modified this equation to include the average pore diameter of the capillary wick. Based on the Lucas-Washburn equation, Fries and Dreyer^21^ (who formulated the Fries-Dreyer equation) ignored the inertial force and used a mathematical rearrangement of the Lambert *W* function to derive the relationship between the height of liquid rise in a capillary wick and its porosity and pore diameter.

Cai et al.^22^ obtained the analytical expression for capillary rise as a function of time by introducing tortuosity and fractal dimension for the tortuous capillary based on the Lucas-Washburn equation, and Cheng et al.^23^ proposed a theoretical model for predicting the one-dimensional movement of water into a single air-filled fracture within a porous media, which replaced a single tube radius with an effective diameter in the model of Cai^22^. Shen et al.^24^ presented a model for calculating capillary rise accounting for the sticky layer and the capillary radius by taking the sticky layer resistance and the capillary radius into the classical Lucas-Washburn equation. Feng et al.^25^ revisited the Lucas-Washburn equation and further considered the effective viscosity and slippage and developed a model of the capillary rise in nanopores. Wang et al.^26^ obtained the modified theoretical equation by incorporating the effect of slip length, dynamic contact angle, and effective viscosity into the Lucas-Washburn equation for the unsaturated transport of CsCl solutions through the C-S-H nanochannel. More recently, Wang et al.^27^ and Chen et al.^28^ studied the capillary water absorption phenomenon in porous ceramic materials, and discussed the mechanism influencing saturation, inclination angle, and pore-structure parameters (including the porosity and particle diameter of the capillary wick) on the rise height. The experimental results showed the same trend for rise height with rise time as described by the Fries-Dreyer equation, yet with a large gap between the two results.

This work seeks to further clarify the relationship between the transport performance of a capillary wick and its pore structure, based on the experimental results as reported by^27^, by establishing a mathematical and physical model to describe the internal transport phenomena in a capillary wick using the COMSOL software platform. The model is then verified by comparing the numerically simulated results with those experimental results^27^. Once verified, the model is used as a basis for determining the influence of saturation, inclination angle, and pore-structure parameters (including porosity, particle diameter, and tortuosity in the capillary wick) on the rise height, velocity, mass, and equilibrium height. Our modified Fries-Dreyer equation provides an improved theoretical basis for research, evaluation, and design of electric vehicle battery refrigerant cooling systems based on capillary wicks.

## Physical and mathematical models of capillary transport

The transport performance of a capillary wick determines the cooling performance of a battery pack cooling system. The rise height and velocity of liquid in a capillary wick are determined mainly by the capillary driving force, viscous resistance, pressure force, and gravity^29^.

### Physical model

**Figure 1:**
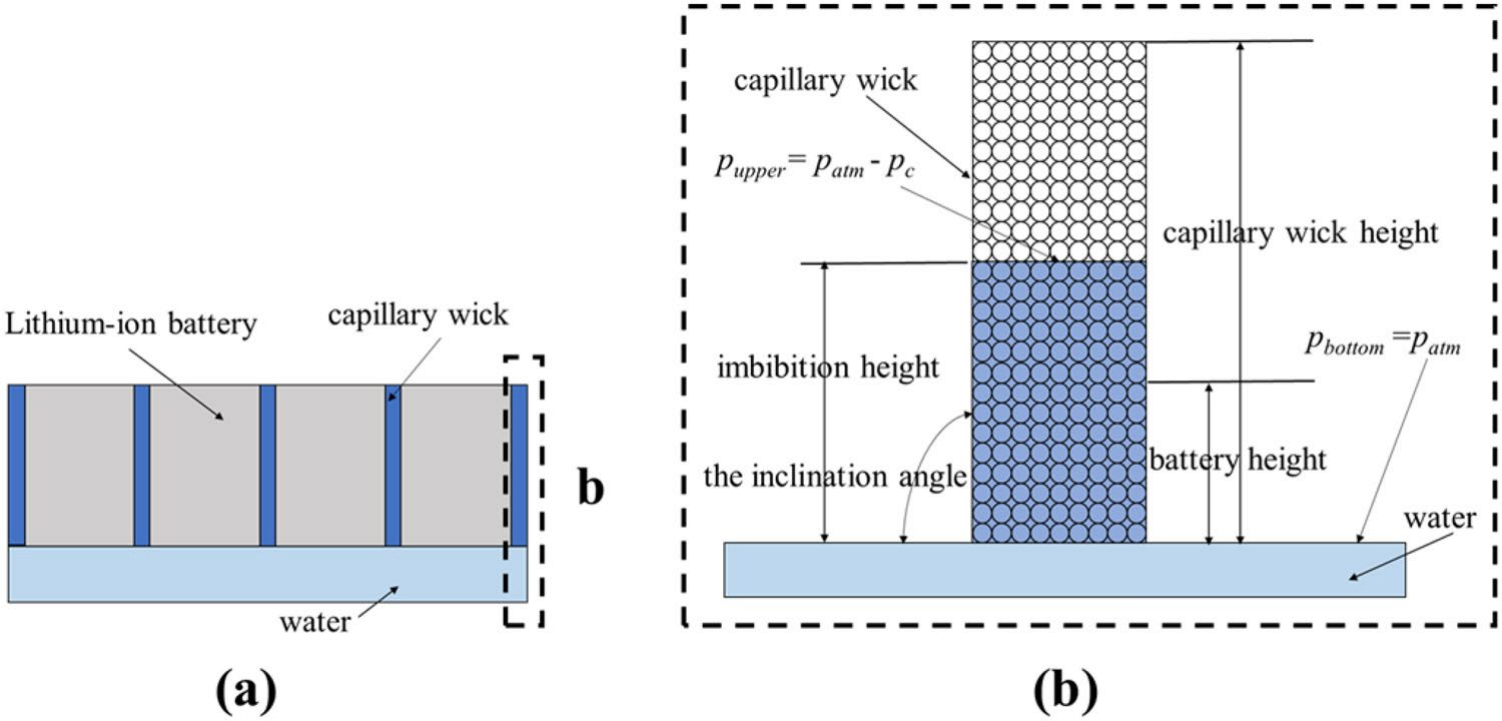
(**a**) Schematic of the direct cooling model of the battery pack; (**b**) schematic of the capillary wick.

In this work, the capillary wick separates the battery, and both are directly immersed into the liquid, as shown in Fig. 1a. An unconsolidated capillary wick with spherical particles is used as a representative example, and water is taken as the liquid. The height, width, and thickness of the capillary wick are 500, 66, and 1mm, respectively, which are based on the battery size (120, 66, and 18 mm, respectively) as given in^30^. To determine the equilibrium height and mass of capillary transport, we vary the height of the capillary wick from 120 mm to 500 mm. To increase the energy density of the battery pack, the thickness of the capillary wick is selected as 1 mm, as shown in Fig. 1b. The capillary wick is placed in the coolant at various inclination angles. Driven by capillary force, the coolant climbs along the long dimension (height) of the capillary wick. When the capillary driving force and gravity reach a balance, the liquid transport has reached its equilibrium height. The following assumptions are made in establishing a mathematical model that suitably implements the transport of liquid in a capillary wick: (1) The capillary wick is treated as homogeneous, rigid, and isotropic. (2) There are no coolant leaks around the capillary wick. (3) The loss of tube viscous resistance follows the Hagen-Poiseuille law.(4) The capillary phenomenon researched here is treated as one dimension. (5) Friction and inertia are neglected. (6) The internal pores in the capillary wick are uniformly connected.

### Mathematical model

The capillary rise of the coolant in the capillary wick can be described by the phase transport equation and Darcy’s law.

#### The phase transport equation

The transport of the wetting/non-wetting phases *i* inside the capillary wick conforms to the following equation that includes the gravity term and the capillary driving force term^31^:

1 $$\begin{aligned} \frac{\partial }{{\partial t}}\left( {{\varepsilon _p}{\rho _i}{s_i}} \right) + \nabla \cdot \left( { - {\rho _i}K\frac{{{K_{r,i}}}}{{{\mu _i}}}\left( {\nabla {p_i} - {\rho _i}{\textbf{g}}} \right) } \right) = 0 \end{aligned}$$

where *t* is time, ${\varepsilon _p}$ refers to porosity; *w* and *n* denote the wetting (water) and nonwetting (air) phases, respectively; *K* is the permeability of the capillary wick; ${\textbf{g}}$ is gravitational acceleration; ${s_i}$, ${K_{r,i}}$, ${\mu _i}$, ${\rho _i}$ and ${p_i}$ are the volume fraction, relative permeability, dynamic viscosity, density, and pressure of the wetting / nonwetting phase, respectively. In Eq. (1),

2 $$\begin{aligned} {s_n}= & {} 1 - {s_w} \end{aligned}$$

3 $$\begin{aligned} K= & {} {{{D^2}{\varepsilon _p}^3} \big /{180{{\left( {1 - {\varepsilon _p}} \right) }^2}}} \end{aligned}$$

where *D* is the particle diameter. The non-wetting phase pressure ${p_n}$ is related to the wetting phase pressure and the capillary driving force ${p_c}({s_w})$ and can be calculated as follows:

4 $$\begin{aligned} {p_n} = {p_c}({s_w}) + {p_w} \end{aligned}$$

The capillary driving force ${p_c}({s_w})$ follows the Brooks-Corey model^31^, which is expressed as:

5 $$\begin{aligned} {p_c}\left( {{s_w}} \right) = \frac{{{{\left( {{{{\varepsilon _p}} \big / K}} \right) }^{\mathrm{{0}}\mathrm{{.5}}}}\sigma J\left( {{s_w}} \right) }}{{{{\left( {{{\left( {{s_w} - {s_{r,w}}} \right) } \big / {\left( {1 - {s_{r,n}} - {s_{r,w}}} \right) }}} \right) }^{{1 \big /{{\lambda _p}}}}}}} \end{aligned}$$

where ${s_{r,w}}$ and ${s_{r,n}}$ are the residual saturation of the wetting and nonwetting phase, respectively, and both values are 0 in this paper; ${\lambda _p}$ is the pore distribution index, and the value is 2 ; $\sigma$ is the surface tension of the wetting phase; $J({s_w})$ is given by:

6 $$\begin{aligned} J({s_w}) = 1.417(1 - {s_w}) - 2.120{(1 - {s_w})^2} + 1.263{(1 - {s_w})^3} \end{aligned}$$

The relative permeability in Eq. (1) follows the Brooks-Corey model^31^, which is calculated as follows:

7 $$\begin{aligned} {K_{r,w}}= & {} {\left( {{{\left( {{s_w} - {s_{r,w}}} \right) } \big / {\left( {1 - {s_{r,n}} - {s_{r,w}}} \right) }}} \right) ^{\left( {{{3 + 2} \big / {{\lambda _p}}}} \right) }} \end{aligned}$$

8 $$\begin{aligned} {K_{r,n}}= & {} {\left( {{{\left( {{s_n} - {s_{r,n}}} \right) } \big / {\left( {1 - {s_{r,n}} - {s_{r,w}}} \right) }}} \right) ^2}\left( {1 - {{\left( {1 - \left( {{{\left( {{s_n} - {s_{r,n}}} \right) } \big /{\left( {1 - {s_{r,n}} - {s_{r,w}}} \right) }}} \right) } \right) }^{{{(1 + 2} \big /{{\lambda _p})}}}}} \right) \end{aligned}$$

The boundary conditions for phase transport are that (1) the mass flux of the bottom and surrounding area on the interface of the capillary wick are 0, and (2) the top mass flux is calculated by the pressure gradient of Darcy’s law. Lagrange multipliers are then used to calculate the mass flux ${q_{{s_w}}}$ of the top air:

9 $$\begin{aligned} {q_{{s_w}}} = {\lambda \big / d} \end{aligned}$$

where *d* is the thickness of the capillary wick. $\lambda$ is the Lagrange multipliers, which are equal to per length and full revolution in axially symmetric models^32^.

#### Darcy’s law

The rise process of the coolant in the capillary wick follows the mass conservation equation, which is expressed as follows:

10 $$\begin{aligned} \frac{\partial }{{\partial \mathrm{{t}}}}\left( {{\varepsilon _p}\overline{\rho }} \right) + \nabla \cdot \left( {\overline{\rho }{\textbf{u}}} \right) = 0 \end{aligned}$$

where the average density of liquid and gas phases is $\overline{\rho }= {s_n}{\rho _n} + {s_w}{\rho _w}$; ${\textbf{u}}$ is the permeation velocity of the two-phase, and follows Darcy’s law, which gives the viscosity resistance term as:

11 $$\begin{aligned} {\textbf{u}} = - \frac{K}{{\overline{\mu }}}\nabla p \end{aligned}$$

where *p* is the pressure; $\overline{\mu }$ is the average viscosity of the liquid and gas phases, and the calculation formula is as follows:

12 $$\begin{aligned} \overline{\mu }= \frac{{\overline{\rho }}}{{{{{K_{r,n}}{\rho _n}} \big /{{\mu _n}}} + {{{K_{r,w}}{\rho _w}} \big /{{\mu _w}}}}} \end{aligned}$$

By inputting Eqs. (11) into (10), the Darcy’s Law interface combines the Darcy’s law with the mass conservation equation as:

13 $$\begin{aligned} \frac{\partial }{{\partial \mathrm{{t}}}}\left( {{\varepsilon _p}\overline{\rho }} \right) + \nabla \cdot \overline{\rho }\left[ - \frac{K}{{\overline{\mu }}}\nabla p\right] = 0 \end{aligned}$$

The boundary conditions are shown in Fig. 1, and there is no flow around. The bottom pressure ${p_{bottom}}$ is the atmospheric pressure ${p_{atm}}$, and the top pressure ${p_{upper}}$ is the atmospheric pressure minus capillary driving force, expressed as follows:

14 $$\begin{aligned} {p_{upper}} = - {p_c}({s_w})f({s_n}) - {\rho _n}{L_0}\textit{g} \end{aligned}$$

where $f({s_n})$ is the smooth step function for increasing the convergence with a range between 0 and 1; ${L_0}$ is the height of the water in the capillary wick.

## Verification of the model

**Figure 2:**
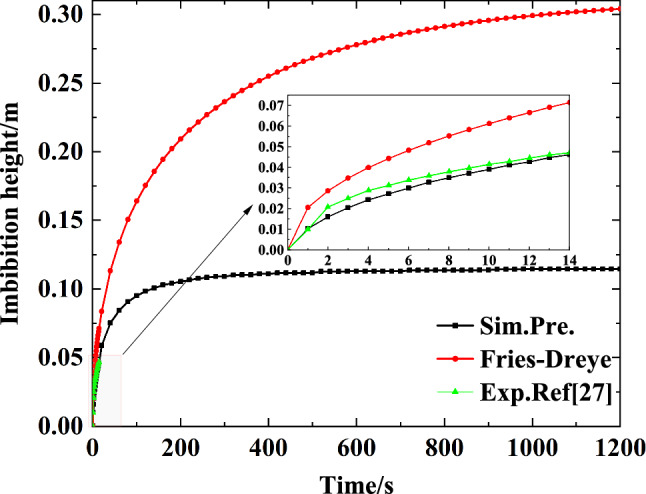
Grid independent test for the model.

The water transport process in the capillary wick is studied by numerical simulation using two modules integrated in COMSOL Multiphysics (version 5.6), a commercial software product based on finite-element methods; one was the module implementing Darcy’s law, and the other models multiphase flow in porous media. To identify the proper grid number for accurate results, rigorous mesh independence trials are conducted. A particular case of mesh independence study is shown in Fig. 2, which presents the numerical results of the mass and equilibrium height simulation according to the grid number. Simulation results are obtained under the grid number of 200 for the capillary wick. The time-dependent solver with a fixed tolerance limit of 0.005 is deployed to solve for the multiphase flow in porous media. As there are very steep gradients in the volume fractions of wetting and non-wetting portions of the wick, an initial time step of 0.0001 is used to make sure that resolution is guaranteed adequate, and the time-step scheme is adaptive, so efficiency is maintained. Table 1 lists some essential parameters used for the model calculation; these parameters are assumed to not change with temperature.

**Table 1:** Material properties of water, air, and the capillary wick at 25 $\mathrm{{^\circ C}}$.

Variable	Parameter	Value	Variable	Parameter	Value
${\rho _w}$	Density of water	1000 $\mathrm{{kg/}}{\mathrm{{m}}^\mathrm{{3}}}$	*D*	Particle diameter	$2 \times {10^{ - 4}}$ m
${\rho _n}$	Viscosity of air	1 $\mathrm{{kg/}}{\mathrm{{m}}^\mathrm{{3}}}$	${s_w}$	Saturation	0
${\varepsilon _p}$	Porosity	0.47	$\psi$	Inclined angle	$90^\circ$
${\mu _n}$	Viscosity of air	$1.76 \times {10^{ - 5}}$ $\mathrm{{pa}} \cdot \mathrm{{s}}$	$\tau$^33^	Tortuosity	1.58
${\mu _w}$	Viscosity of water	0.001 $\mathrm{{pa}} \cdot \mathrm{{s}}$	*L*	Length	0.5 m
$\sigma$	Surface tension of water	0.0724 N/m	*Wi*	Width	0.066 m
${\lambda _p}$	Pore size distribution index	2	*d*	Thickness	0.001 m

**Figure 3:**
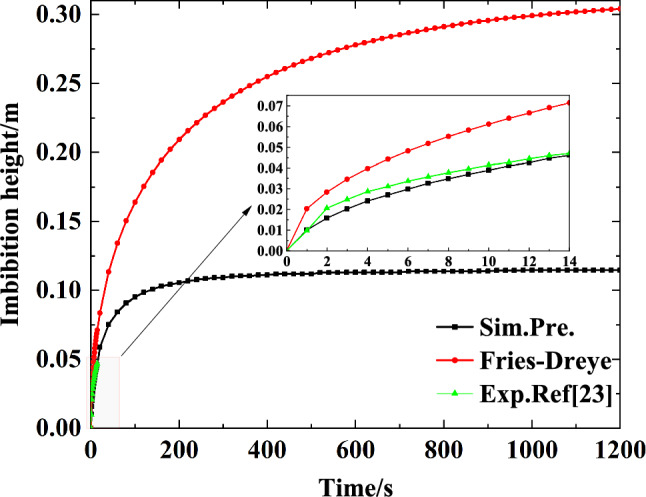
Comparison of simulated results of the present model with model calculation results and experimental data from ref.^27^.

Figure 3 graphs the rise height over time in the capillary wick as calculated by the simulation and compares it with the results of the Fries-Dreyer equation and with the experimental results obtained by citeWang2021Capillary. As shown in Fig. 3, the rise height of the liquid in the capillary wick increases with time but eventually stabilizes. This trend is similar to the results of the Fries-Dreyer equation and the experimental data, but our simulated results are in better agreement with the experimental results than the Fries-Dreyer results. In the time period 0 to 14 s, the mean relative error between the simulated results and the Fries-Dreyer results is 47.50 $\%$; the mean relative error for the simulation is 5.19 $\%$.

In this work, the numerically simulated results are fitted to improve the accuracy of the theoretical results derived with the Fries-Dreyer equation. Thus, the coefficients *c* and *d* are introduced to modify the dimensionless numbers Eh (equilibrium height number) and Tn (capillary time number), as follows:

15 $$\begin{aligned} h(t) = c \times \mathrm{{Eh}}\left[ {1 + W\left( { - {e^{ - 1 - d \times \mathrm{{Tn}}}}} \right) } \right] \end{aligned}$$

where *W* is the Lambert function^21^; the dimensionless number Eh indicates that, under infinite time, the height gain converges into a maximum value. The expression for Eh is:

16 $$\begin{aligned} \mathrm{{Eh}} = \frac{\alpha }{\beta } = \frac{{\sigma J\left( {{s_w}} \right) }}{{{\rho _w}\textit{g}\sin \psi }}{\left( {{{{\varepsilon _p}} \big / K}} \right) ^{0.5}} \end{aligned}$$

The dimensionless number Tn defines the time when the rise height reaches 99$\%$ of the equilibrium height. It is given by

17 $$\begin{aligned} \mathrm{{Tn}} = t\frac{{{\beta ^2}}}{\alpha } = \frac{{t{\rho _w}^2{\textit{g}^2}K{{\sin }^2}\psi }}{{\sigma J\left( {{s_w}} \right) {\tau ^2}}}{\left( {{K \big / {{\varepsilon _p}}}} \right) ^{0.5}} \end{aligned}$$

The main methods for curve fitting include least-squares method, best first approximation method, data and gradient descent method, etc. The principle of least-squares method is to find the minimum sum of squares of errors to determine the best matching function corresponding to the data, which can intuitively express the functional relationship between the dependent variable and the independent variable. The advantage of the least-squares method is that it does not require the objective function to pass through data points and only requires the objective function to reach an approximate degree of discrete points^34^. Therefore, the least-squares method is used to fit the numerical simulation results. The coefficients *c* and *d* can be calculated by curve fitting the simulated data by the least-squares method, using a sum-of-squared-errors of $2.594 \times {10^{ - 5}}$ and an R-square of 0.9915. The fitting results can be obtained by applying Eq. (15) to this case, obtaining:

18 $$\begin{aligned} h(t) = 0.419\mathrm{{Eh}}\left[ {1 + W\left( { - {e^{ - 1 - 3.068\mathrm{{Tn}}}}} \right) } \right] \end{aligned}$$

## Analysis of results

In addition to pore-structure parameters, such as the particle diameter of the capillary wick, the porosity and tortuosity affect the transport performance. The saturation of the liquid and the inclination angle of the capillary wick also directly affect the transport performance. In this section, we discuss the mechanisms by which these parameters influence the transport performance of the capillary wick.

### The effect of saturation

**Figure 4:**
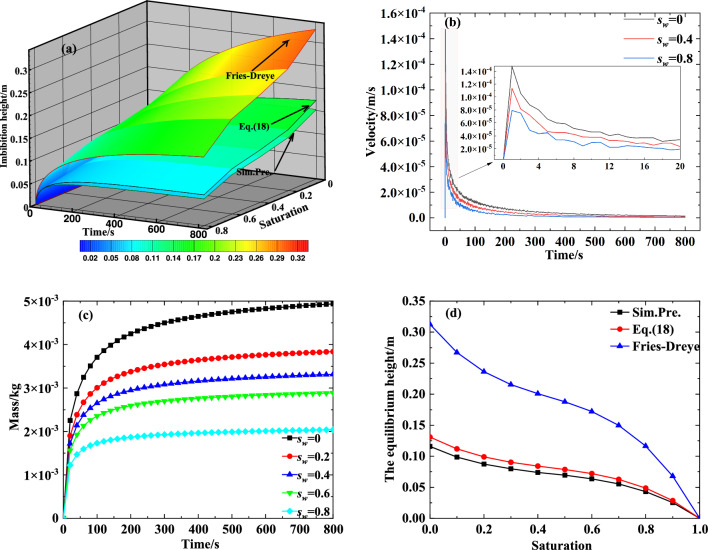
The influence of saturation on the transport performance of a capillary wick: (**a**) Comparison diagram of the influence of saturation on the height of the liquid rise; (**b**) The influence of saturation on the velocity of the liquid rise; (**c**) The influence of saturation on the mass of the liquid rise; (**d**) Comparison diagram of the influence of saturation on the equilibrium height of the liquid rise.

To evaluate the influence of saturation on the transport performance of the capillary wick, we simulated the process of liquid rising in a vertical capillary wick (particle diameter 0.2 mm, porosity 0.47, and tortuosity 1.58) using various saturations. The results are shown in Fig. 4a–d. Figure 4a illustrates that, according to Eqs. (5) and (6), the liquid capillary driving force decreases with an increase in saturation, which reduces the height of the liquid rise in the capillary wick. This result is consistent with the change trend of the Fries-Dreyer equation and with Eq. (18). However, Eq. (18) provides results closer to the numerically simulated results.

The influence of saturation on the velocity and mass of the liquid rise based on the numerically simulated results is shown in Fig. 4b and c, and the comparison diagram of the influence of saturation on the equilibrium height of the liquid rise is shown in Fig. 4d. Figure 4b shows that the velocity of the fluid rise increases sharply and then decreases, and the larger the saturation, the smaller the velocity of the fluid rise. The results shown in Fig. 4c,d together reveal that as saturation increases, the height and mass of the fluid rise in the capillary wick monotonically decreases; thus, higher saturation is not conducive to meeting the supply demand for refrigerant when the power battery pack is at a high temperature or fast charging. Figure 4d shows that, regardless of the saturation, the calculated results of Eq. (18) are closer than the Fries-Dreyer equation to the numerically simulated results.

### The effect of inclination angle

**Figure 5:**
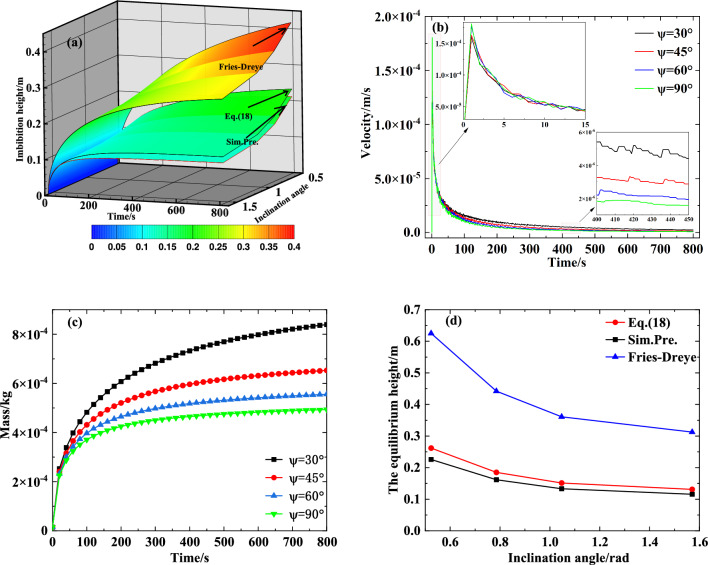
The influence of inclination angle on the transport performance of a capillary wick: (**a**) Comparison diagram of the influence of inclination angle on the height of the liquid rise; (**b**) The influence of inclination angle on the velocity of the liquid rise; (**c**) The influence of inclination angle on the mass of the liquid rise; (**d**) Comparison diagram of the influence of inclination angle on the equilibrium height of the liquid rise.

To evaluate the influence of the inclination angle on the transport performance of the capillary wick, we simulated the process of liquid rising in an inclined capillary wick (saturation 0, particle diameter 0.2 mm, porosity 0.47, and tortuosity 1.58) using various inclination angles. The results are shown in Fig. 5a–d.

Figure 5a illustrates that, as the inclination angle increases, the height of the liquid rise in the capillary wick decreases. The reason is that when the inclination angle decreases, the component of gravity also decreases, while other forces remain unchanged, which causes the height of the liquid rise to increase gradually. This result is consistent with the change trend of the Fries-Dreyer equation and Eq. (18). However, Eq. (18) provides results closer to the numerically simulated results.

The influence of inclination angle on the velocity and mass of the liquid rise based on the numerically simulated results is shown in Fig. 5b,c, and the comparison diagram of the influence of inclination angle on the equilibrium height of the liquid rise is shown in Fig. 5d. Figure 5b shows that in the early stages, the larger the inclination angle, the greater the velocity of the fluid rise, and, with the rise of the climbing liquid, the larger the inclination angle, the smaller the velocity of the fluid rise. The results shown in Fig. 5c,d together reveal that as inclination angle increases, the height and mass of the fluid rise in the capillary wick monotonically decrease. Thus, higher inclination angle is not conducive to meeting the supply demand for refrigerant when the power battery pack is at a high temperature or fast charging. Figure 5d shows that, regardless of the inclination angle, the calculated results of Eq. (18) are closer than the Fries-Dreyer equation to the numerically simulated results.

### The effect of the pore structure parameters

The pore-structure parameters of the capillary wick include porosity, particle diameter, and tortuosity. These parameters directly affect the transport performance of the capillary wick, as shown by the Fries-Dreyer equation and Eq. (18).

#### The effect of particle diameter

**Figure 6:**
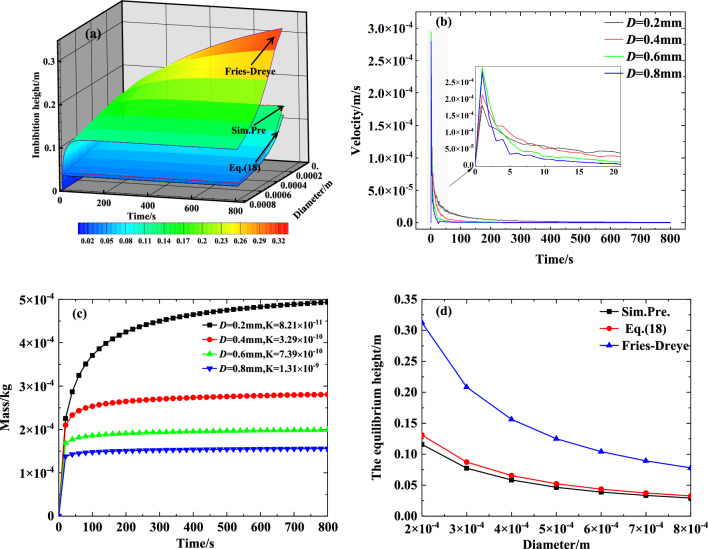
The influence of particle diameter on the transport performance of a capillary wick: (**a**) Comparison diagram of the influence of particle diameter on the height of the liquid rise; (**b**) The influence of particle diameter on the velocity of the liquid; (**c**) The influence of particle diameter on the mass of the liquid rise; (**d**) Comparison diagram of the influence of particle diameter on the equilibrium height of the liquid rise.

To evaluate the influence of particle diameter on the transport performance of the capillary wick, we simulated the process of liquid rising in a vertical capillary wick (saturation 0, porosity 0.47, and tortuosity 1.58) using various particle diameters. The results are shown in Fig. 6a–d.

Figure 6a shows that as the particle diameter increases, its internal pore structure increases, and the capillary driving force decreases, so the height of the liquid rise in the capillary wick steadily decreases. This finding is consistent with the change trend of the Fries-Dreyer equation and with Eq. (18). However, Eq. (18) provides results closer to the numerically simulated results.

The influence of the particle diameter on the velocity and mass of the liquid rise based in the numerically simulated results is shown in Fig. 6b and c, and the comparison diagram of the influence of the particle diameter on the equilibrium height of the liquid rise is shown in Fig. 6d. Figure 6b shows that in the early stages, the larger the particle diameter, the greater the velocity of the fluid rise, and, with the rise of the climbing liquid, the larger the particle diameter, the smaller the velocity of the fluid rise. The results shown in Fig. 6c,d together reveal that as particle diameter increases, the height and mass of the fluid rise in the capillary wick monotonically decrease. Thus, larger particle diameter is not conducive to meeting the supply demand for refrigerant when the power battery pack is at a high temperature or fast charging. Figure 6d shows that, regardless of particle diameter, the calculated results of Eq. (18) are closer than the Fries-Dreyer equation to the numerically simulated results.

#### The effect of porosity

**Figure 7:**
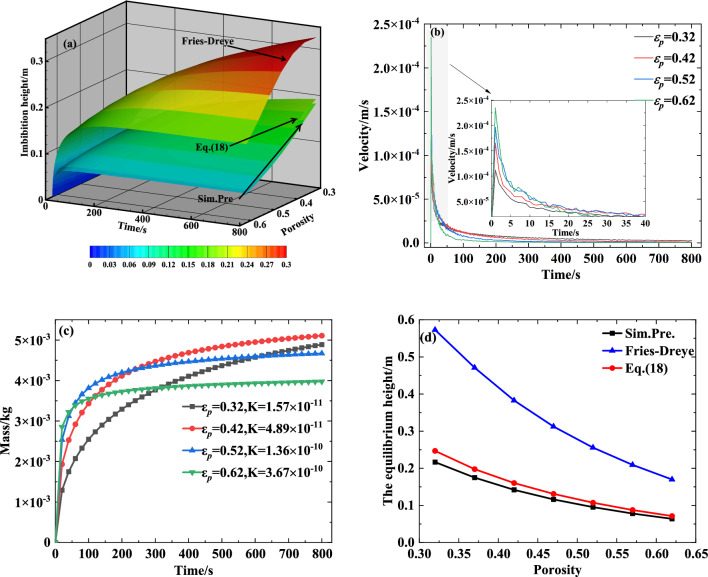
The influence of porosity on the transport performance of a capillary wick: (**a**) Comparison diagram of the influence of the porosity on the height of the liquid rise; (**b**) The influence of porosity on the velocity of the liquid rise; (**c**) The influence of porosity on the mass of the liquid rise; (**d**) Comparison diagram of the influence of porosity on the equilibrium height of the liquid rise.

To evaluate the influence of porosity on the transport performance of the capillary wick, we simulated the process of liquid rising in a vertical capillary wick (saturation 0, particle diameter 0.2 mm, and tortuosity 1.58) using various porosities. The results are shown in Fig. 7a–d.

Figure 7a illustrates that, as the porosity increases while the particle diameter remains unchanged, the derivation of the equation is less than 0 when substituting Eq. (3) into (5). So the capillary driving force decreases, which reduces the height of liquid rise in the capillary wick. This is consistent with the change trend of the Fries-Dreyer equation and with Eq. (18). However, Eq. (18) provides results closer to the numerically simulated results.

The influence of the porosity on the velocity and mass of the liquid rise based on the numerically simulated results is shown in Fig. 7b and c, and the comparison diagram of the influence of the porosity on the equilibrium height of the liquid rise is shown in Fig. 7d. Figure 7c shows that in the early stages, the larger the porosity, the greater the initial transport mass of the fluid; however, at later times, the larger porosities limit the total mass transfer. The reason is that, as shown in Fig. 7b, in the early stage, the larger the porosity, the greater the velocity of the fluid rise, so the greater the transport mass of the fluid. With the rise of the climbing liquid, the larger the porosity, the smaller the velocity of the fluid rise, which limits the total transport mass of the fluid. Figure 7d shows that with an increase in the porosity, the equilibrium height of the fluid rise in the capillary wick steadily decreases, which is not conducive to meeting the supply demand for refrigerant when the power battery pack is at a high temperature or fast charging. Figure 7d shows that, regardless of the porosity, the calculated results of Eq. (18) are closer than the Fries-Dreyer equation to the numerically simulated results.

#### The effect of tortuosity

The internal structure of the capillary wick is complex, and the internal flow path of its liquid is winding rather than straight. It can be described by tortuosity, which is defined as the ratio of the actual length ${L_f}$ that the flow travels to the straight-line length ${L_s}$ of the capillary wick^22^:

19 $$\begin{aligned} \tau = {{{L_f}} \big / {{L_s}}} \end{aligned}$$

**Figure 8:**
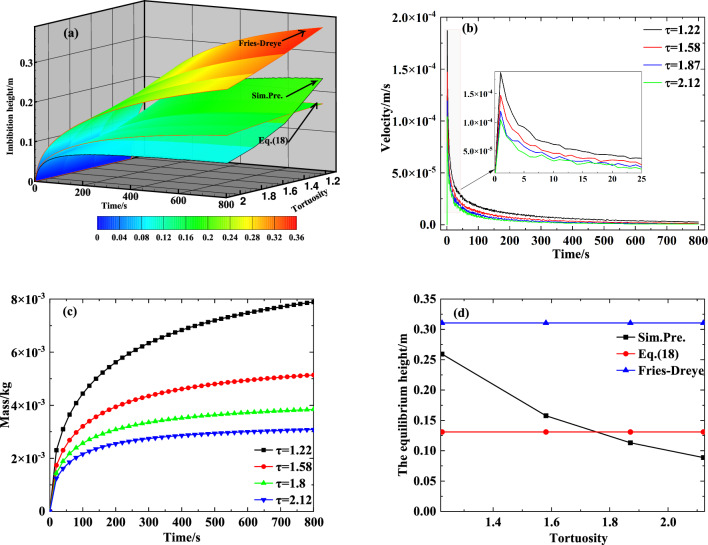
The influence of tortuosity on the transport performance of capillary wick: (**a**) Comparison diagram of the influence of tortuosity on the height of the liquid rise; (**b**) The influence of tortuosity on the velocity of the liquid rise; (**c**) The influence of tortuosity on the mass of the liquid rise; (**d**) Comparison diagram of the influence of tortuosity on the equilibrium height of the liquid rise.

To evaluate the influence of tortuosity on the transport performance of the capillary wick, we simulated the process of liquid rising in a vertical capillary wick (saturation 0, particle diameter 0.2 mm, and porosity 0.47) using various tortuosities. The results are shown in Fig. 8a–d. Figure 8a illustrates that, as the tortuosity increases, according to Eq. (19), the actual length ${L_f}$ increases, while the straight-line length ${L_s}$ in the capillary wick steadily decreases. This result is consistent with the change trend of the Fries-Dreyer equation and with Eq. (18). However, Eq. (18) provides results closer to the numerically simulated results. The influence of the tortuosity on the velocity and mass of the liquid rise based on the numerically simulated results is shown Fig. 8b and c and a comparison diagram of the influence of the tortuosity on the equilibrium height of the liquid rise is shown in Fig. 8d.

Figure 8b shows that the larger the tortuosity, the smaller the velocity of the fluid rise. The results shown in Fig. 8c,d together reveal that as tortuosity increases, the rise height and transport mass of the fluid in the capillary wick monotonically decrease; thus, higher tortuosity is not conducive to meeting the supply demand for refrigerant when the power battery pack is at a high temperature or fast charging. Figure 8d shows that, regardless of tortuosity, the theoretical value of the equilibrium height is not affected by the tortuosity, mainly because neither the Fries-Dreyer equation nor Eq. (18) include tortuosity. When the tortuosity is in the range of 1.4–2.0, Eq. (18) is closer than the Fries-Dreyer equation to the numerically simulated result; this range is similar to the range of 1.44–1.58 proposed by^35^.

## Discussion

**Figure 9:**
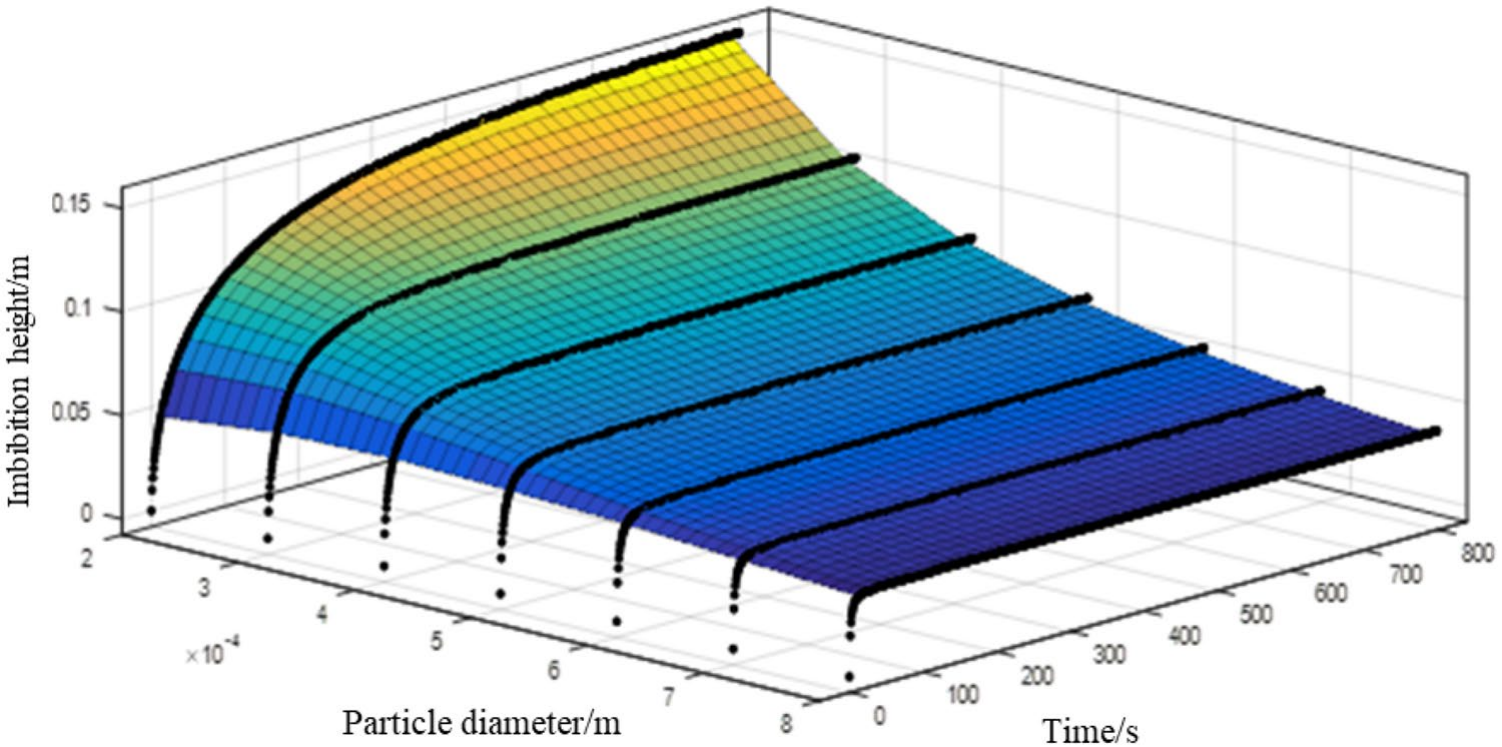
Imbibition height of the simulated predicted values in the capillary wick at different diameter.

Although the results which Eq. (18) gives are closer to the experimental results than those of the Fries-Dreyer equation, there is still some mismatch with the experimental results, as shown in Figs. 4a, 5a, 6a, 7a and 8a. One reason is that the sample size used to fit Eq. (18) is too small. To improve the accuracy of the fitting formula, the numerically simulated results of the transport process in the capillary wick were taken as samples under various combinations of saturation, inclination angle, particle diameter, porosity, and tortuosity. Based on the Fries-Dreyer equation, the least squares method was used to fit the simulated data. The results are shown in Fig. 9; the regression formula is:

20 $$\begin{aligned} h(t) = 0.3785\frac{\alpha }{\beta }\left[ {1 + W\left( { - {e^{ - 1 - {{3.982{\beta ^2}t} \big / \alpha }}}} \right) } \right] \end{aligned}$$

Table 2 lists the mean relative error ${\varepsilon ^1}$, ${\varepsilon ^2}$ and ${\varepsilon ^3}$ for different saturation, inclination angle, and pore structure parameters using the Fries-Dreyer equation, Eqs. (18, 20) and the numerical simulated results, respectively. It can be seen that ${\varepsilon ^2}$ is much lower than ${\varepsilon ^1}$, while ${\varepsilon ^3}$ is smaller yet, showing that the accuracy of the fitted Eq. (20) (after including simulated data in the least-squares analysis) is higher. Table 2 shows that for saturation, inclination angle, particle diameter, and porosity, ${\varepsilon ^3}$ has less than ${\varepsilon ^2}$ and all of the ${\varepsilon ^3}$ are less than 5$\%$. When the tortuosity is 1.22, 1.87 and 2.12, ${\varepsilon ^2}$ and ${\varepsilon ^3}$ is more than 20$\%$. When the tortuosity is 1.58, ${\varepsilon ^2}$ and ${\varepsilon ^3}$ decrease from 7.9$\%$ to 2.4$\%$, and the accuracy is higher, which is consistent with a tortuosity of 1.58 proposed in^33^.

**Table 2:** The mean relative error between the different theoretical model value and the simulated predicted values during different saturation, inclined angle, porosity, tortuosity and particle diameter.

Parameters	Value	${\varepsilon ^1}/\%$	${\varepsilon ^2}/\%$	${\varepsilon ^3}/\%$
$\mathrm{{Saturation}}\left( {{s_w}} \right)$	0	88.64	7.91	2.50
	0.2	99.37	10.07	3.58
	0.4	105.80	11.16	4.05
	0.6	111.98	12.09	4.42
	0.8	127.29	13.83	4.96
$\mathrm{{Angle}}\,\left( \psi \right)$	$30^\circ$	74.26	4.32	0.42
	$45^\circ$	98.72	9.60	3.10
	$60^\circ$	114.39	11.87	3.93
	$90^\circ$	125.22	12.93	4.16
$\mathrm{{Diameter}}\,\left( D \right)$	$2 \times {10^{ - 4}}$	125.22	12.93	3.35
	$3 \times {10^{ - 4}}$	157.06	13.93	3.70
	$4 \times {10^{ - 4}}$	165.29	13.70	3.78
	$5 \times {10^{ - 4}}$	166.88	13.09	3.80
	$6 \times {10^{ - 4}}$	167.84	12.95	3.82
	$7 \times {10^{ - 4}}$	169.16	13.23	3.82
	$8 \times {10^{ - 4}}$	167.76	12.49	3.82
$\mathrm{{Porosity}}\,\left( {{\varepsilon _p}} \right)$	0.32	60.82	0.93	1.38
	0.37	79.94	6.00	1.55
	0.42	102.45	10.39	3.56
	0.47	125.21	12.92	4.15
	0.52	144.18	13.77	3.89
	0.57	156.76	13.86	3.44
	0.62	163.27	13.59	2.91
$\mathrm{{Tortuosity}}\,\left( \tau \right)$	1.22	44.23	23.26	28.49
	1.58	88.63	7.90	2.40
	1.87	125.08	34.11	28.80
	2.12	156.89	57.17	52.18

## Conclusion

The influences of saturation, inclination angle, and pore-structure parameters on capillary transport performance were studied by numerical calculation and simulation in this work. Using the Fries-Dreyer equation and the simulation values as benchmarks, a new theoretical formula describing the transport performance of a capillary wick was obtained by modifying the dimensionless numbers Eh and Tn. The main conclusions are as follows.

1. A mathematical and physical model of the transport process in a capillary wick was built using the COMSOL software platform. The numerically simulated results of this work were closer to the experimental results than those of the Fries-Dreyer equation. The results of the fitted Eq. (18) were closer than those of the Fries-Dreyer equation to the experimental results.
2. Analyzing the influence of saturation and inclination angle on the transport performance of a capillary wick indicated that the imbibition height, mass, and equilibrium height of the capillary wick all increased with a decrease in saturation or inclination angle. Regardless of the saturation or inclination angle, the fitting by Eq. (18) was closer to the numerically simulated results than was the Fries-Dreyer equation.
3. Analysis of the influence of pore-structure parameters, such as particle diameter, porosity, and tortuosity, on the imbibition performance of the capillary wick showed that the imbibition height, mass, and equilibrium height of the capillary wick all decreased with the rise of particle diameter, porosity, and tortuosity.
4. The calculated results of fitting with Eq. (18) were closer to the simulated results than were the results of the Fries-Dreyer equation. However, the mean relative error between Eq. (18) and the simulated values was still substantial. To improve the accuracy of the fitted formula, the numerically simulated results of the transport process in the capillary wick under various saturation, inclination angle, particle diameter, porosity, and tortuosity were taken as samples and the equation was re-fitted based on the Fries-Dreyer equation. This new fitted equation, Eq. (20) provides results where the maximum deviation from the simulated results did not exceed 5$\%$ when the tortuosity is 1.58. The modified theoretical model developed in this work shows significant potential for revealing the influence of various values of the several considered parameters on the imbibition performance of the capillary wick.

## Data Availability

The datasets used and/or analysed during the current study available from the corresponding author on reasonable request.
